# Supplementary material for: Modulation of peroxisomal import by the PEX13 SH3 domain and a proximal FxxxF binding motif
Source: Nat Commun. 2024 Apr 18;15:3317. doi: 10.1038/s41467-024-47605-w (PMC11024197; doi:10.1038/s41467-024-47605-w)
Supplement: Supplementary file 1 — Supplementary Information [file 41467_2024_47605_MOESM1_ESM.pdf]

Supplementary Material

Modulation of peroxisomal import by the PEX13 SH3 domain and a proximal FxxxF binding motif

Stefan Gaussmann[1,2§], Rebecca Peschel[3§], Julia Ott[3], Krzysztof M. Zak[2], Judit Sastre[4], Florent Delhommel[1,2], Grzegorz M. Popowicz[1,2], Job Boekhoven[4], Wolfgang Schliebs[3], Ralf Erdmann[3*], Michael Sattler[1,2*]

Contents

Supplementary Figures
Supplementary Figure 1 2
Supplementary Figure 2 3
Supplementary Figure 3 4
Supplementary Figure 4 5
Supplementary Figure 5 6
Supplementary Figure 6 7
Supplementary Figure 7 8
Supplementary Figure 8 9
Supplementary Figure 9 11
Supplementary Figure 10 13
Supplementary Figure 11 14
Supplementary Figure 12 15
Supplementary Tables 17
Supplementary Table 1 17
Supplementary Table 2 18
Supplementary Table 3 18
Supplementary Table 4 18
Supplementary Table 5 19
Supplementary Table 6 20
Supplementary Table 7 21
Supplementary Table 8 22
Supplementary Table 9 23
References 24

Supplementary Figure 1

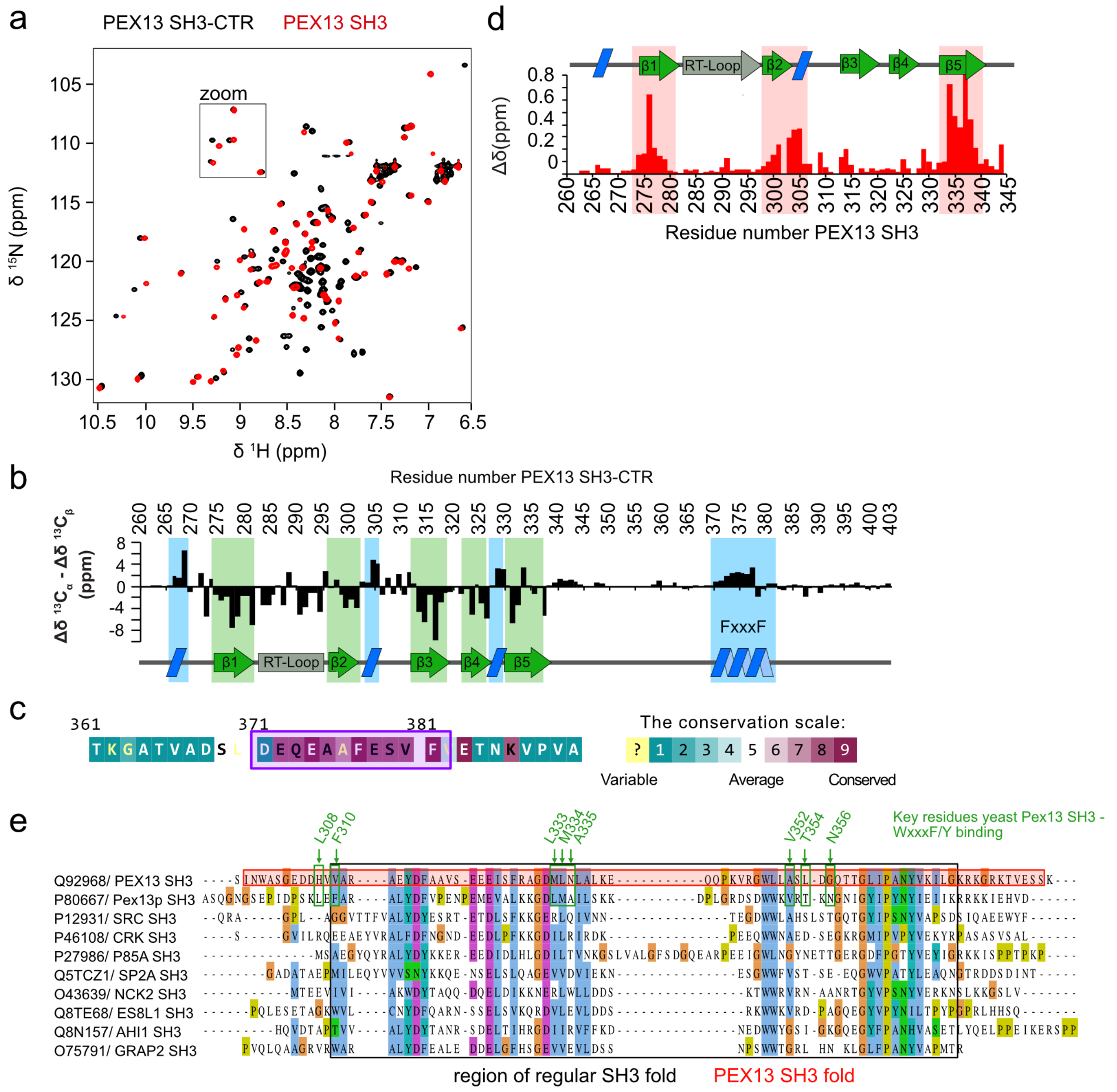

Supplementary Fig. 1. Conformation and NMR analysis of human PEX13 C-terminus. (a) Overlaid 2D spectra of PEX13 SH3-CTR (black) and PEX13 SH3 (red). A zoomed-view is shown in **Fig. 1 (b)** ^{13}C secondary chemical shifts ($\Delta\delta^{13}C_{\alpha}$ - $\Delta\delta^{13}C_{\beta}$) of PEX13 SH3-CTR (261-403) support the typical β-sandwich fold of the SH3 domain and the presence of a short α-helical motif comprising the FxxxF motif. **(c)** Sequence alignment with 186 mammalian PEX13 sequences plotted with ConSurf web server [1-3] shows high conservation of the FxxxF motif (purple box). **(d)** chemical shift perturbations plotted on the PEX13 SH3 sequence with secondary structure indicated on top. **(e)** Sequence alignment of human PEX13 SH3 with yeast PEX13 SH3 and SH3 domains from human SRC, CRK, AHI1, NCK2, ES8L1, GRAP2, P85A, and SPD2A (with known structures). The sequence alignment was done with the ± 10 residues flanking the SH3 fold. The black box indicates the regular SH3 fold and the red box the complete fold of human PEX13 SH3. Green arrows indicate key residues for yeast Pex13 SH3 / WxxxF/Y binding. Note that those residues are poorly conserved from yeast to human.

Supplementary Figure 2

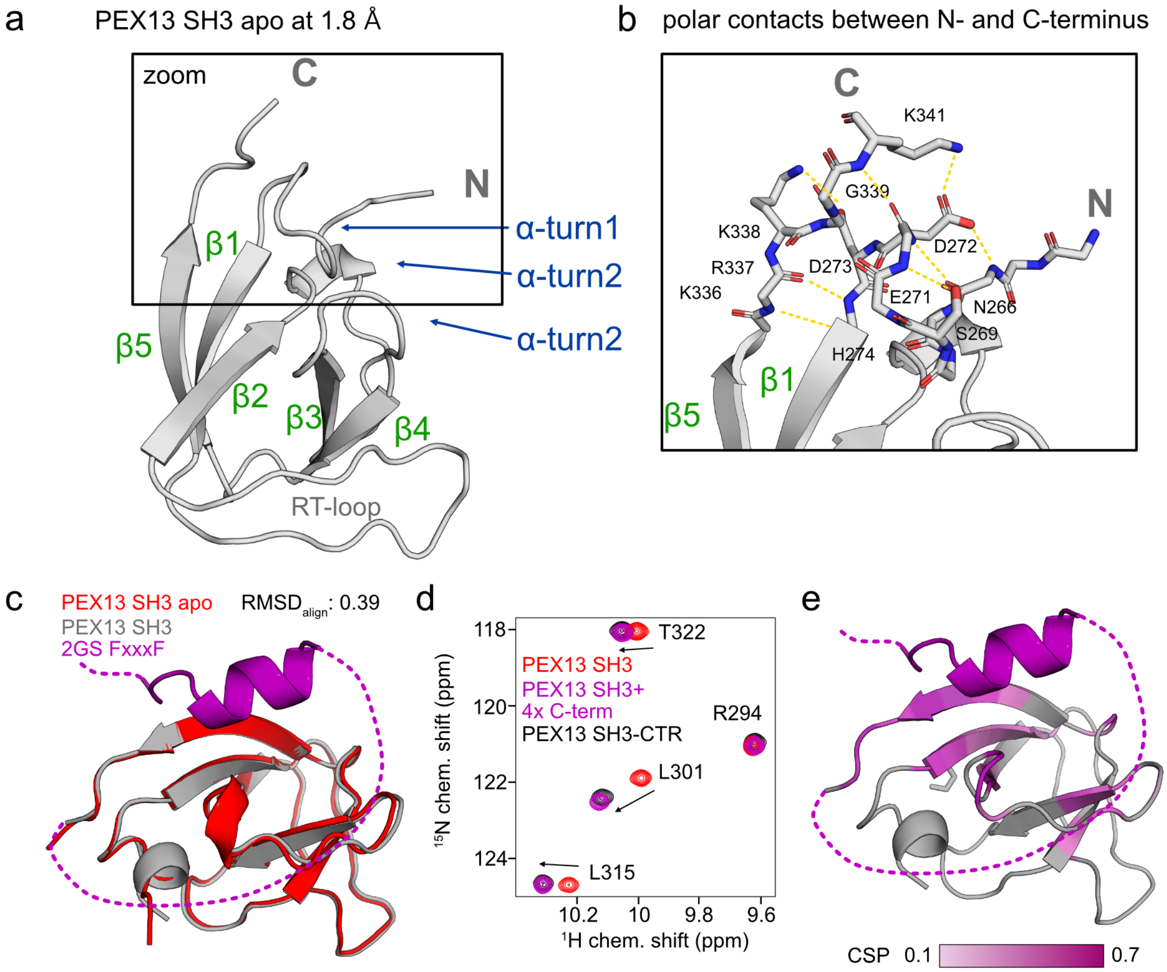

Supplementary Fig. 2. Structural features of apo PEX13 SH3 and confirmation of PEX13 SH3 – FxxxF structure in solution. (a) Apo structure of PEX13 SH3 solved with 1.8 Å resolution. **(b)** Zoomed-view showing a network of hydrogen bonds in the on the N – and C-terminal regions (yellow). **(c)** Superimposition of the structures PEX13 SH3 (red) and PEX13 SH3 (gray) in complex with FxxxF motif (purple). **(d)** Overlay of ^{1}H, ^{15}N correlation spectra of PEX13 SH3 (red) PEX13 SH3-CTR (black) and PEX13 SH3 titrated with FxxxF peptide (350-403) (purple). PEX13 SH3 titrated with FxxxF peptide represents the native spectrum of PEX13 SH3-CTR. **(E)** Chemical shift perturbations from PEX13 SH3 / FxxxF titration mapped on the PEX13 SH3-FxxxF complex structure.

Supplementary Figure 3

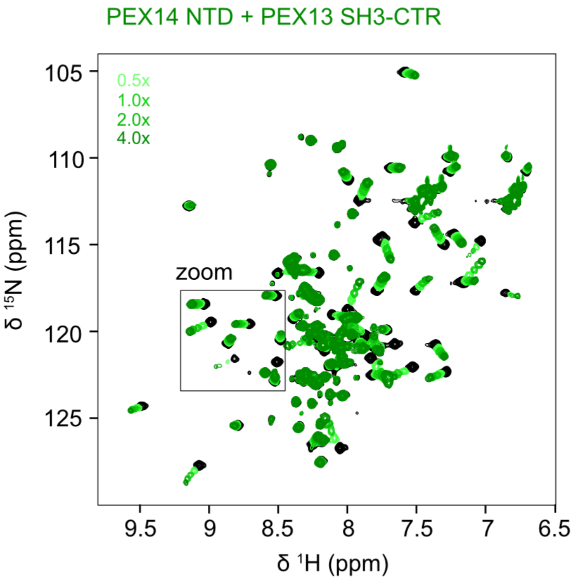

Supplementary Fig. 3. Titration of ^{15}N PEX14 NTD with PEX13 SH3-CTR. Overlaid 2D spectra of PEX14 NTD (black) titrated with increasing concentrations PEX13 SH3-CTR (261-403) (green scale). Zoom is shown in **Fig. 3a**.

Supplementary Figure 4

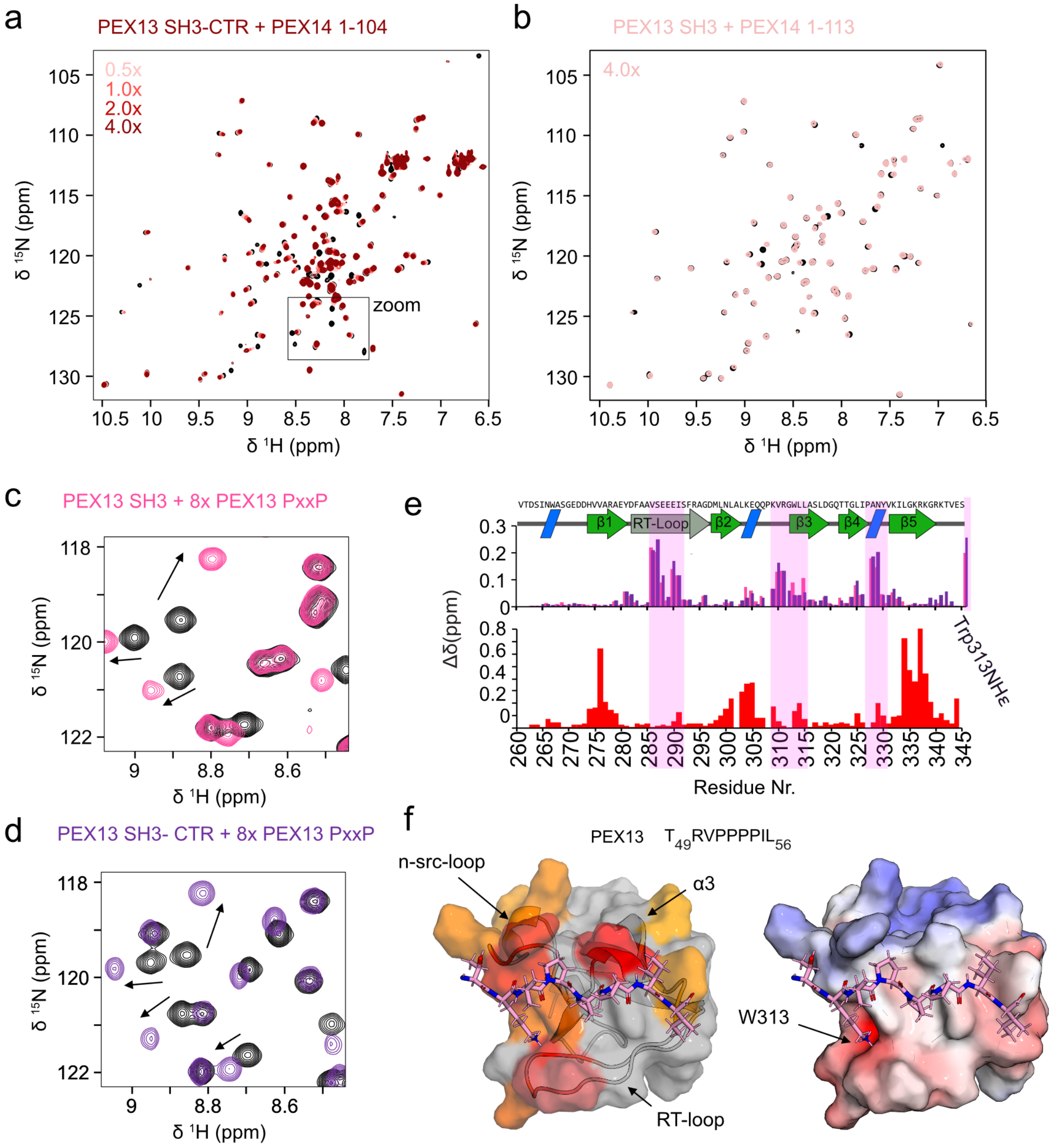

Supplementary Fig.4. Interactions of PEX13 SH3 with PxxP peptides. (a) Overlaid 2D spectra of PEX13 SH3-CTR (black) titrated with increasing concentrations PEX14 NTD (1-104) (red scale). Zoom is shown in **Fig. 4a**. **(b)** 2D spectra of PEX13 SH3 with 4x excess of PEX14 NTD_{long} (1-113) **(c)** 2D spectra of PEX13 SH3 (black) with 8x excess of PEX13 PxxP peptide (49-61) (pink). **(d)** 2D spectra of PEX13 SH3-CTR (black) with 8x excess of PEX13 PxxP peptide (49-61) (dark purple) **(e)** Chemical shift perturbations from PxxP titration on SH3 (pink) and SH3-CTR (dark purple) (upper panel) and from FxxxF titration on SH3 (red/bottom panel) mapped on the PEX13 SH3 sequence demonstrating non-overlapping binding sites **(F)** Structure of PEX13 SH3 with modelled PEX13 PxxP (49-56) represented with transparent surface showing chemical shift perturbations (yellow to red gradient) from NMR titrations (left panel) and electrostatic surface (right panel). PxxP sequence shown above. Binding of PEX13 PxxP (49-61) induces typical CSPs in the regions of RT-loop, n-src-loop and α3 (α10) [4].

Supplementary Figure 5

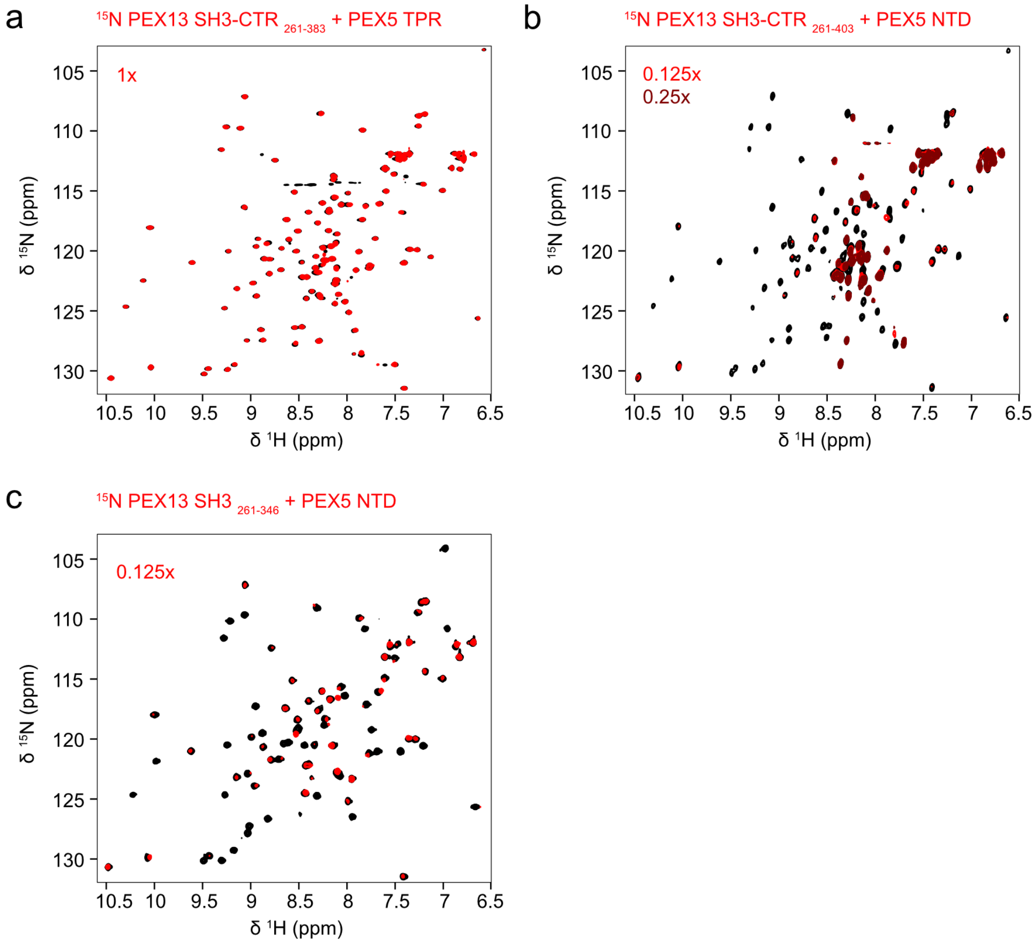

Supplementary Fig.5. Titration of ^{15}N PEX13 SH3-CTR or PEX13 SH3 with PEX5 TPR or PEX5 NTD. (a) Overlaid 2D spectra of PEX13 SH3-CTR (261-383; missing the last 20 amino acids; black) titrated with equimolar concentration of PEX5 TPR domain (red). **(b)** Overlaid 2D spectra of PEX13 SH3-CTR (black) titrated with 0.125x (red) or 0.25x (dark red) excess of PEX5 NTD. Notably, resonances experience excessive line-broadening at 0.125 (1/8) ligand concentration. **(c)** Overlaid 2D spectra of PEX13 SH3 (black) titrated with 0.125x (red) excess of PEX5 NTD. The SH3 titration experiment shows very similar line-broadening effect as seen with PEX13 SH3-CTR indicating binding of PEX5 NTD to PEX13 SH3.

Supplementary Figure 6

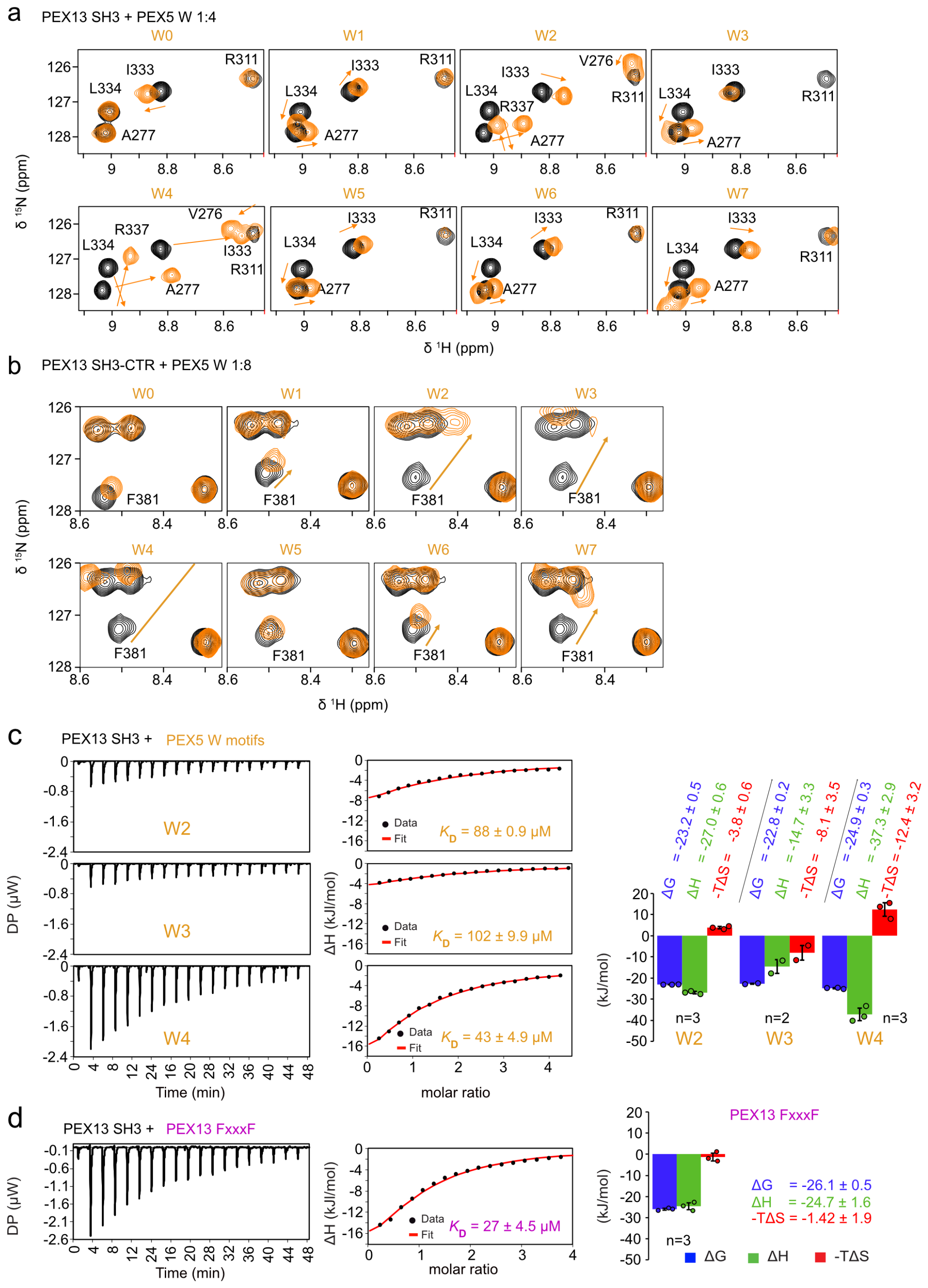

Supplementary Fig. 6. (Competitive) binding of PEX13 SH3 and SH3-CTR with PEX5 W peptides. Average values and error bars (SD) are calculated from 3 different experiments (n=3). **(a)** Overlaid 2D spectra of PEX13 SH3 (black) and with 4x excess of PEX5 (di)romatic peptides (orange). **(b)** Overlaid 2D spectra of PEX13 SH3-CTR (black) and with 8x excess of PEX5 (di)aromatic peptides (orange). **(c)** ITC titration of PEX13 SH3 with PEX5 W2, W3 and W4 **(d)** ITC titration of PEX13 SH3 with FxxxF peptide (350-403).

Supplementary Figure 7

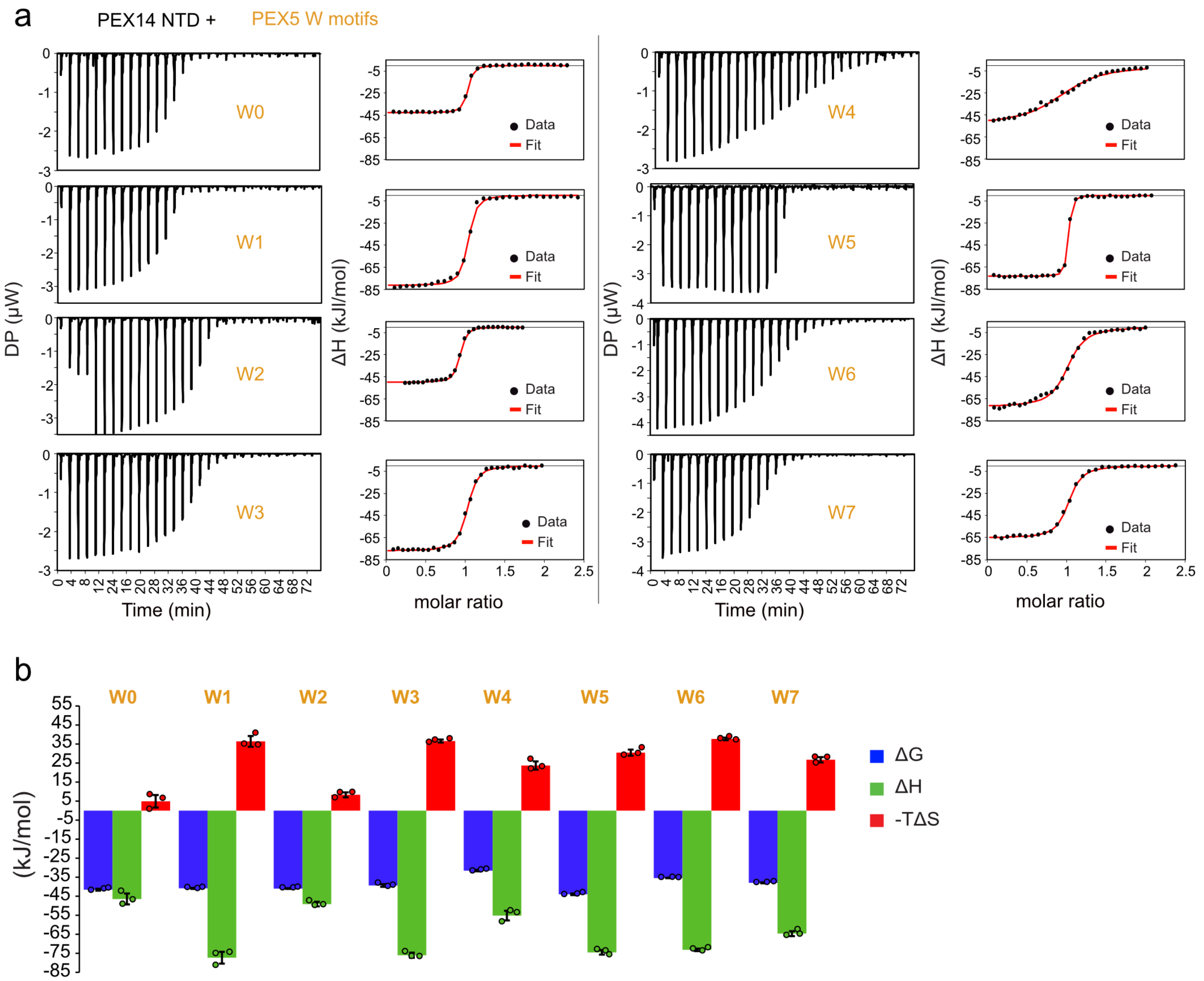

Supplementary Fig. 7. Isothermal titration calorimetry of PEX14 NTD/ PEX5 W interactions. (a) ITC titration of PEX14 NTD (1-104) with PEX5 (di)aromatic peptide motifs. **(b)** Energetic contribution of ITC titration PEX14 NTD (1-104) with PEX5 (di)aromatic peptide motifs. Average values and error bars (SD) are calculated from 3 different experiments (n=3).

Supplementary Figure 8

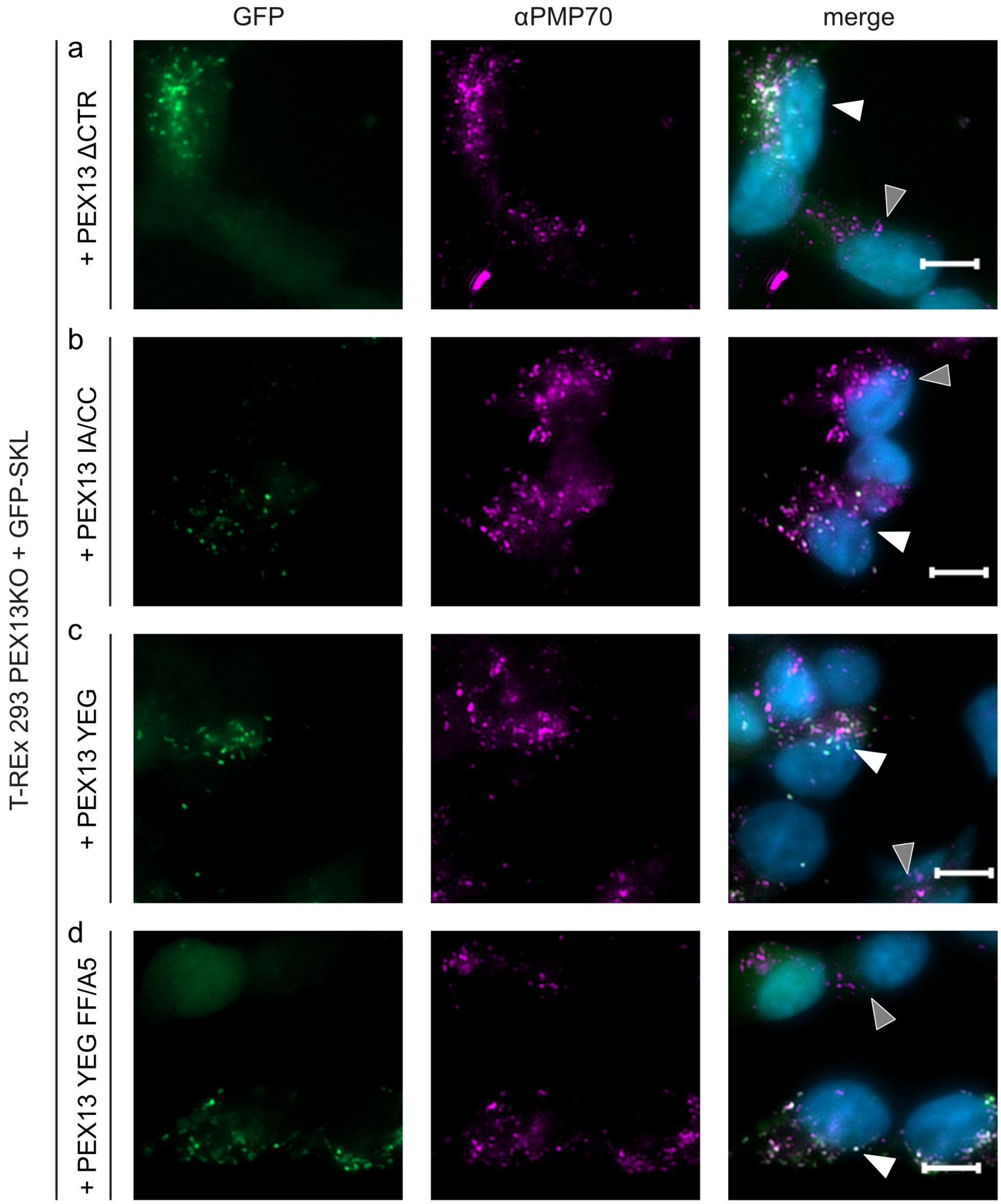

Supplementary Fig. 8. PEX13 SH3-proximal FxxxF motif modulates PTS1 import. TREx 293 cells and PEX13-deficient (PEX13 KO) T-REx 293 cells were transfected with bicistronic expression plasmids encoding GFP-PTS1 and different PEX13 truncation and mutation variants as indicated on the left. Rescue of the PTS1 import defect of PEX13 KO cells was monitored by fluorescence microscopy with GFP-SKL as reporter (left panels). Detection of peroxisomal membranes was achieved via PMP70 specific antibody indicated by magenta fluorescence punctate pattern (middle panels). Overlap of green and magenta dots in the merge (right panels) represents functional matrix import. Nuclei were stained with DAPI (blue, right panels). For each experiment, one cell showing no peroxisomal protein import is marked with a gray

triangle and one cell showing import is marked by a white triangle, if present. Scale bar: 5 µm. In one representative experiment a total of 1216 cells was analyzed. **(a)** Expression of PEX13 missing the full C-terminal region (ΔCTR) in PEX13 KO cells does not rescue PTS1 import similar to the expression of the ΔSH3 mutant. **(b)** Transfection with a PEX13 double cysteine mutant where the FxxxF motif is locked on the SH3 domain under oxidative conditions (PEX13 IA/CC) led to a larger but not significant reduction of the import efficiency. Expression of PEX13 mutants incapable of (di)aromatic penta peptide binding with **(c)** or without functional FxxxF motif **(d)** show a similar reduced import efficiency.

Supplementary Figure 9

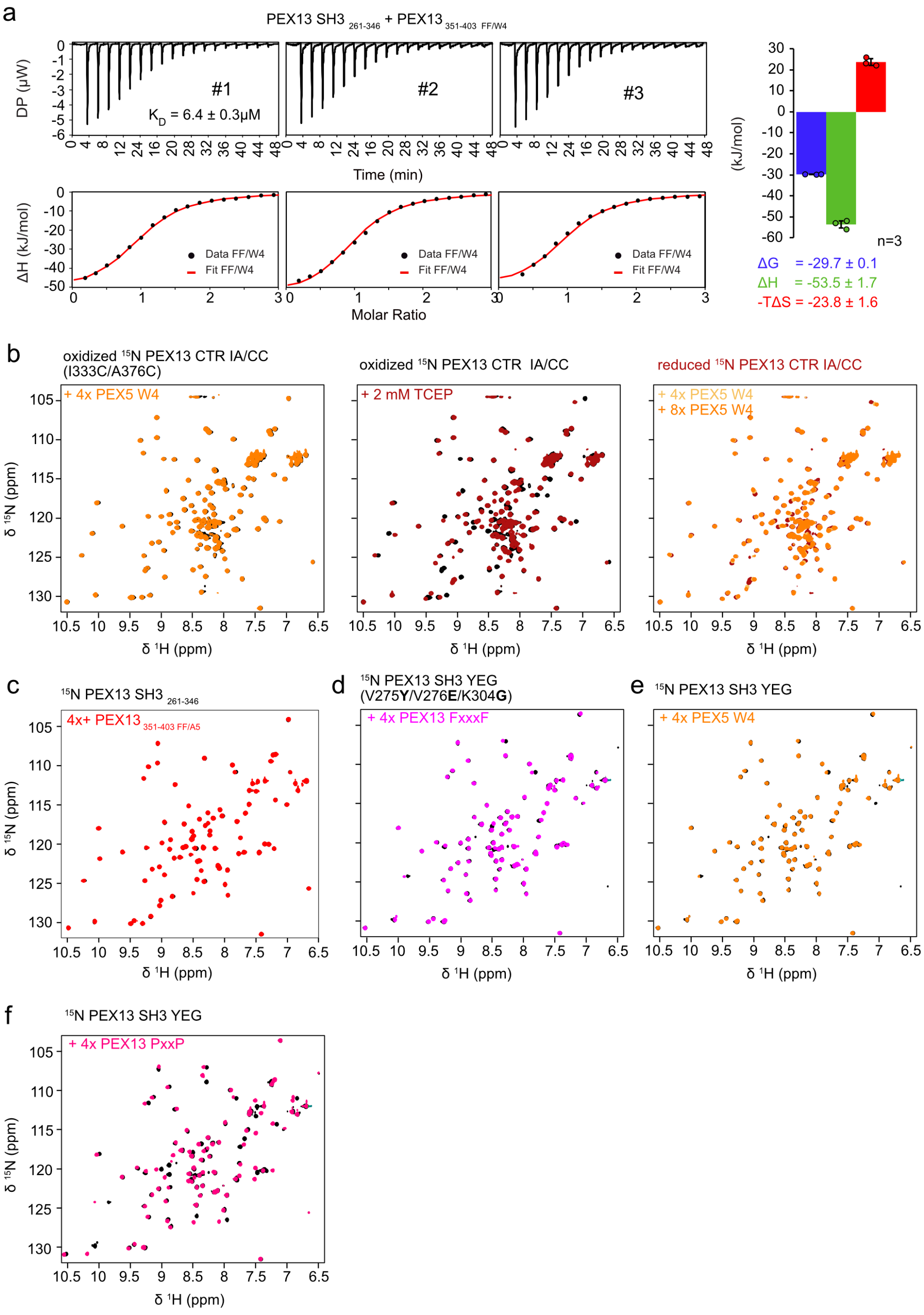

Supplementary Fig. 9. PEX13 mutants and their molecular interactions with FxxxF, WxxxF/Y and PxxP peptides. (a) ITC experiments with the SH3 domain and a PEX13 C-term carrying the FxxxF to PEX5 W4 mutation (FF/W4) show a clear binding event with a dissociation constant $K_D \sim 6$ µM. The curve was fitted to a one binding site model with N=1. Average values and error bars (SD) are calculated from 3 different experiments (n=3). **(b, left)** NMR titration of PEX5 W4 to oxidized ^{15}N PEX13-CTR double cysteine mutant I333C/A376C (IA/CC) does not show binding. **(b, middle)** Reduction of ^{15}N

PEX13-CTR IA/CC shows partial opening of the FxxxF binding indicated by spectral changes. **(b, right)** NMR titration of PEX5 W4 to reduced ^{15}N PEX13-CTR IA/CC shows binding of the PEX5 W4 motif to the SH3 domain indicated by chemical shift changes. **(c)** Overlaid 2D spectra of PEX13 SH3 (black) titrated with 4x (red) excess of PEX13 C-terminal peptide (351-403) with the FxxxF motif mutated to AAAAA (FF/AA) indicate no binding activity. **(d)** NMR titration of ^{15}N labeled PEX13 SH3 triple mutant V275Y/V276E/K304G (YEG) with 4x PEX13 FxxxF peptide and **(e)** 4x PEX5 W4 peptide show no interaction while titration with 4x PEX13 PxxP peptide **(f)** shows binding.

Supplementary Figure 10

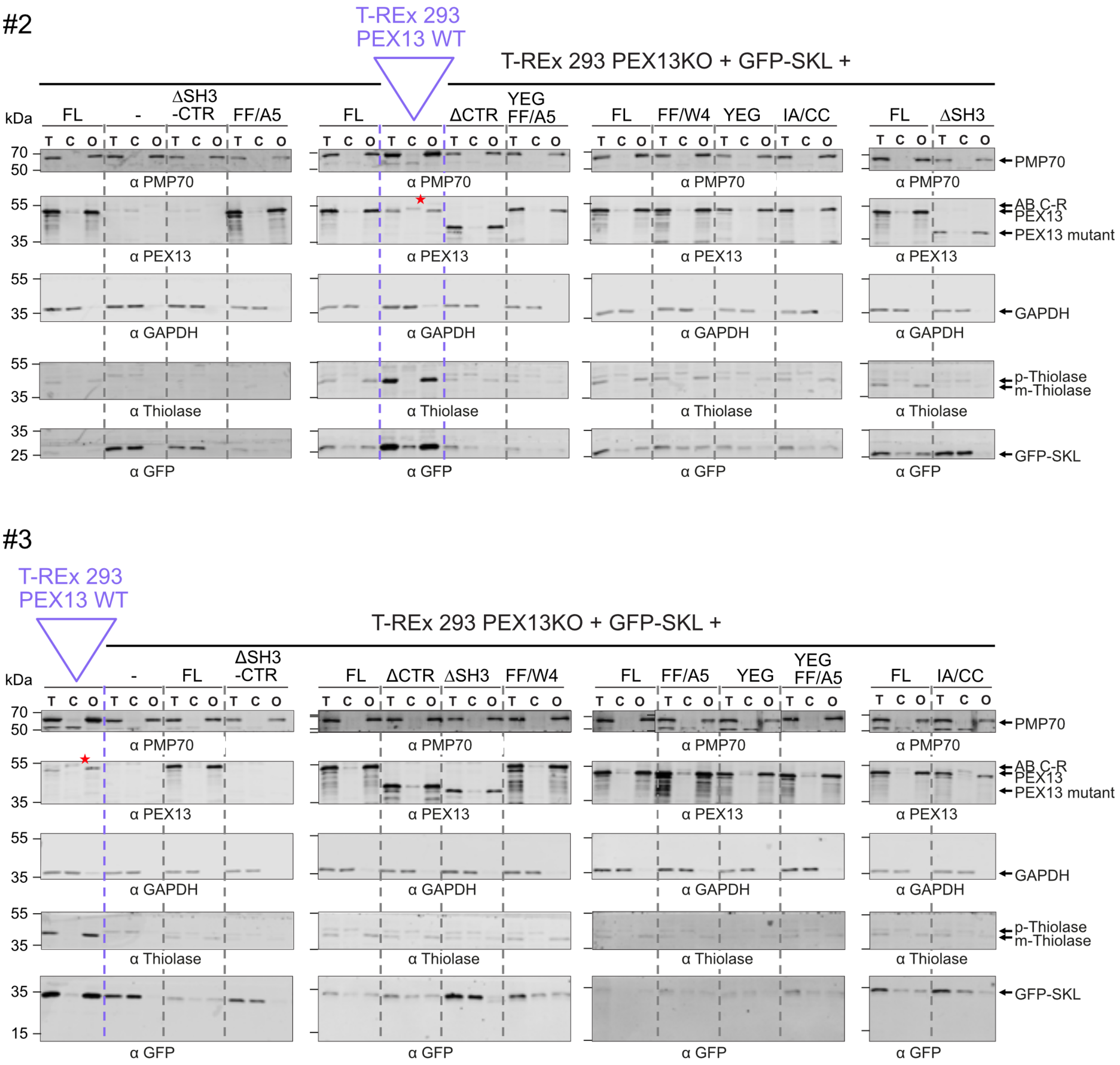

Supplementary Fig.10. Replicates of the fractionation experiment. T-REx 293 cells transfected with GFP-SKL and T-REx 293 PEX13KO cells transfected with a bicistronic vector encoding different PEX13 constructs and GFP-SKL were used for the preparation of total lysates (T) and for fractionation of the cell into cytosol and organelles (C and O, respectively). As readout served the localization of GFP representing PTS1 import. A PEX13 antibody cross-reaction with a protein >55kDa is indicated as AB C-R and red stars. Additionally, PTS2 import can be monitored by detecting Thiolase, which exists in a cytosolic pre-form (p-Thiolase) and in a mature, processed form (m-Thiolase) when imported into the peroxisome. All immunoblots shown are from different gels and from one biological replicate. PMP70 and GAPDH serve as loading controls for the organellar and cytosolic fraction, respectively. Expression of PEX13 variants and GFP-SKL was verified with the corresponding antibodies. PEX13 ΔSH3-CTR is not recognized by the PEX13 antibodies used.

Supplementary Figure 11

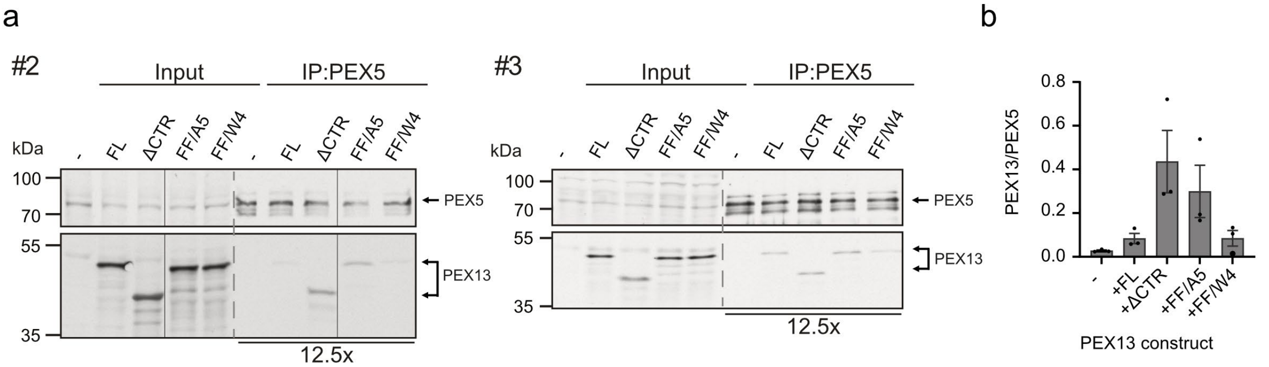

Supplementary Fig. 11. Repetitions of PEX13 pull-down experiments (#2 and #3). a) PEX13 FL, PEX13 ΔCTR and PEX13 harboring the indicated mutations FxxxF to A5 and FxxxF to W4 were expressed in T-REx 293 PEX13 KO cells. The cell lysates were subjected to immunoprecipitation with PEX5 antibody and analyzed by immunoblotting. **b)** The signal intensities of pulled-down PEX5 and PEX13 were measured using the ImageJ software. Black dots indicate individual data points from each biological replicate. Average values and standard error of the mean (SEM) are derived from three independent experiments (n=3).

Supplementary Figure 12

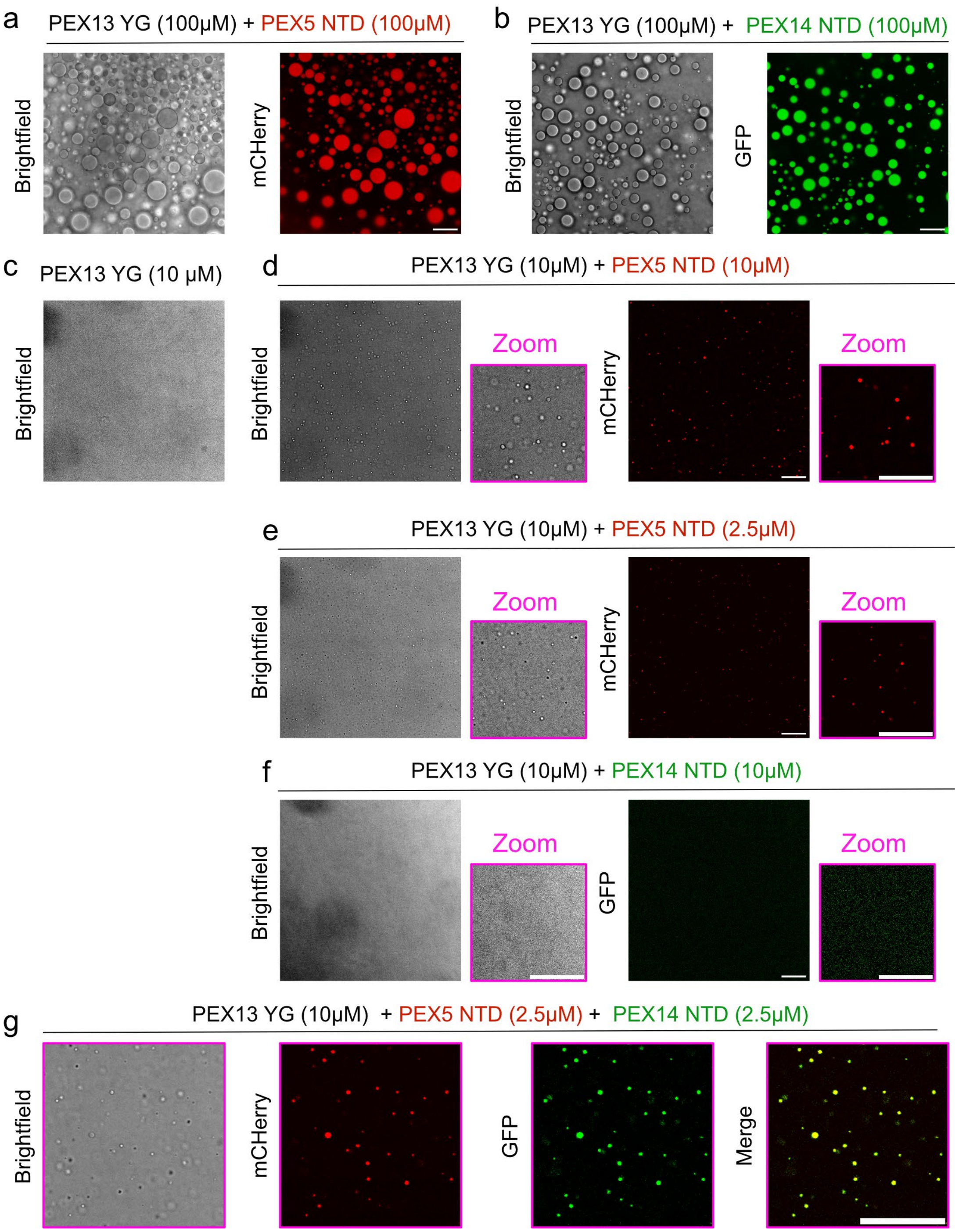

Supplementary Fig. 12. Condensation of PEX13 YG with simultaneous partition of PEX5 NTD and PEX14 NTD. Condensation experiments with PEX13 YG (His-SUMO-PEX13 40-120), PEX5 NTD (1-330) mCherry, PEX14 NTD (1-104) and His-SUMO were performed in 50 mM Tris pH 7.5, 100 mM NaCl and 10% PEG8000 at RT. Scale bars indicate 25µm. Zoomed views in magenta boxes. Experiments were conducted as technical replicates. **(a)** PEX13 YG forms large condensates at 100µM allowing simultaneous partition of PEX5 NTD (mCherry). **(b)** PEX13 YG forms large condensates at 100µM allowing simultaneous partition of PEX14 NTD (GFP) **(c)** PEX13 YG does not form condensates at 10

μM. **(d)** 10 μM and **(e)** 2.5μM PEX5 NTD promotes condensate formation with 10 μM PEX13 YG**. (f)** 10 μM PEX14 NTD does not promote condensate formation with 10 μM PEX13 YG. **(g)** PEX14 NTD can simultaneously partition with PEX5 NTD promoted PEX13 YG condensates at low concentration.

Supplementary Tables

Supplementary Table 1

Crystallographic data for PEX13 SH3, PEX13 SH3 2GS FxxxF and PEX13 SH3 W4 structures.

PDB entry	PEX13 SH3<br>7Z0I	PEX13 SH3 2GS FxxxF<br>7Z0J	PEX13 SH3 GS W4<br>7Z0K
Space group	I 41	P 31 2 1	P 31 2 1
Cell parameters (Å, °)	43.94 43.94 86.78<br>90.00 90.00 90.00	86.80 86.80 65.43<br>90.00 90.00 120.00	87.66 87.66 66.11<br>90.00 90.00 120.00
**Data collection**			
Wavelength (Å)	0.999995	1.000029	1.0332
Temperature (K)	100	100	100
Resolution range (Å)	43.39-1.80<br>(1.84-1.80)	49.36-2.3<br>(2.38-2.3)	49.86-2.3<br>(2.38-2.3)
Total no. of observed reflections	104090 (6202)	260870 (25799)	468378 (25836)
Number of unique reflections	7660 (462)	12998 (1250)	13362 (1275)
R_{merge}	0.082 (1.443)	0.282 (2.426)	0.495 (10.645)
R_{pim}	0.034 (0.588)	0.091 (0.774)	0.118 (3.458)
CC1/2	1 (0.738)	0.997 (0.652)	0.992 (0.588)
I/σ(I)	19.7 (2.1)	11.8 (1.6)	17.0 (1.8)
Completeness (%)	99.9 (99.5)	100 (100)	100 (100)
Multiplicity	13.6 (13.4)	20.1 (20.6)	35.1 (20.3)
Wilson B-factor ($Å^2$)	26.2	35.67	45.11
**Refinement**			
Rwork (%)	0.182	0.197	0.193
Rfree (%)	0.229	0.224	0.217
No. Of atoms	619	1326	1359
Ligand atoms	11	12	0
Solvent atoms	51	75	61
**Model quality**			
RMS (Bonds)	0.0087	0.0133	0.0156
RMS (angles)	1.498	1.9	2.05
Ramachandran favored (%)	96.05	97.56	96.93
Ramachandran allowed (%)	3.95	2.44	3.07
Ramachandran outliers	0	0	0
Rotamer outliers (%)	6.35	6.62	10
Clashscore	0.8	2.63	3.71

Supplementary Table 2

Isothermal titration calorimetry of PEX14 NTD with PEX13 SH3-CTR or PEX13 C-terminal peptide (351-403).

Construct	N	K_D (µM)	∆G (kJ/mol)	∆H (kJ/mol)	-T∆S (kJ/mol)
PEX13 SH3-CTR	1	5.4 ± 1.3	-30.2 ± 0.56	-13.4 ± 1.27	-16.7 ± 1.72
PEX13 FxxxF	1	2.8 ± 0.4	-31.8 ± 0.38	-48.3 ± 1.37	16.5 ± 1.74

Supplementary Table 3

Isothermal titration calorimetry of PEX13 SH3 with PEX5 W2, W3, W4 or PEX13 C-terminal peptide (351-403) with WT FxxxF or introduced PEX4 W4 motif.

Peptide	N	K_D (µM)	∆G (kJ/mol)	∆H (kJ/mol)	-T∆S (kJ/mol)
PEX5 W2	1	88.1 ± 0.1	-23.17 ± 0.05	-27.00 ± 0.57	3.81 ± 0.58
PEX5 W3	1	102.2 ± 9.9	-22.80 ± 0.20	-14.65 ± 3.25	-8.14 ± 3.46
PEX5 W4	1	43.4 ± 4.9	-24.93 ± 0.26	-37.30 ± 2.94	-12.38 ± 3.22
PEX13 FxxxF	1	26.9 ± 4.5	-26.13 ± 0.45	-24.73 ± 1.64	-1.42 ± 1.87
PEX13 FxxxF to W4	1	6.4 ± 0.2	-29.67 ± 0.09	-53.50 ± 1.69	23.8 ± 1.61

Supplementary Table 4

Isothermal titration calorimetry of PEX14 NTD with PEX5 W-peptide motifs.

Peptide	N	K_D (nM)	∆G (kJ/mol)	∆H (kJ/mol)	-T∆S (kJ/mol)
PEX5 W0	1	55.1 ± 0.1	-41.53 ± 0.50	-46.43 ± 2.87	4.91 ± 3.28
PEX5 W1	1	72.3 ± 7.0	-40.83 ± 0.26	-77.27 ± 3.07	36.43 ± 2.81
PEX5 W2	1	73.6 ± 5.4	-40.96 ± 0.19	-49.10 ± 1.21	8.33 ± 1.30
PEX5 W3	1	136.5 ± 37.9	-39.33 ± 0.70	-76.00 ± 1.34	36.63 ± 0.78
PEX5 W4	1	3136.7 ± 469.5	-31.50 ± 0.37	-55.17 ± 2.53	23.70 ± 2.18
PEX5 W5	1	20.5 ± 3.6	-43.96 ± 0.42	-74.47 ± 1.19	30.47 ± 1.62
PEX5 W6	1	636.3 ± 21.9	-35.40 ± 0.08	-73.13 ± 0.66	37.70 ± 0.70
PEX5 W7	1	236.0 ± 23.0	-37.90 ± 0.22	-64.63 ± 1.33	26.77 ± 1.39

Supplementary Table 5

Primer list for cloning into pETM13S vector.

Construct	Boundaries	Forward short	Forward tail	Reverse short	Reverse tail
**pETM13S**	backbone	GGAAGCTGAGTTGGCTGCTGCCAC	TAACAAAGCCCGAAAGGAAGCTGAGTTG	GCGGTGAGCCTCAATAATATCGTTATCC	GCCACCAATCTGTTCGCGGTG
**PEX13 YG**	40-120	ATGACCCGTCCGGGTCAG	GAACAGATTGGTGGCATGACCCGTCCGGGTCAGC	CTGCACAAAACGACTAGGGGG	TTTCGGGCTTTGTTACTGCACAAAACGACTAGGGGG
**PEX13 SH3**	261-346	GTTACTGATTCCATCAACTGGGCCTC	GAACAGATTGGTGGCGTTACTGATTCCATCAACTGGGCCTC	TGAGCTTTCTACTGTTTTACGTCCTTTGC	TTTCGGGCTTTGTTATGAGCTTTCTACTGTTTTACGTCCTTTGC
**PEX13 FxxxF**	261-383	GTTACTGATTCCATCAACTGGGCCTC	GAACAGATTGGTGGCGTTACTGATTCCATCAACTGGGCCTC	CTCAACGAAGACGGATTCGAAGGC	TTTCGGGCTTTGTTACTCAACGAAGACGGATTCGAAGGC
**PEX13 SH3-CTR**	261-403	GTTACTGATTCCATCAACTGGGCCTC	GAACAGATTGGTGGCGTTACTGATTCCATCAACTGGGCCTC	TTTGGTACCTCACAAATCCTGTTTTTCACCG	TTTCGGGCTTTGTTATTTGGTACCTCACAAATCCTGTTTTTCACCG
**PEX13 2GSc FxxxF**	chimera	TAACAAAGCCCGAAAGGAAGCTGAGTTG	GGCGGTGGAGGCAGCGGAGGTGGAGGAAGCGACGAGCAAGAAGCCGCCTTCGAATCCGTCTTCGTTGAGTAACAAAGCCCGAAAGGAAGCTGAGTTG	TGAGCTTTCTACTGTTTTACGTCCTTTGC	CTCAACGAAGACGGATTCGAAGGCGGCTTCTTGCTCGTCGCTTCCTCCACCTCCGCTGCCTCCACCGCCTGAGCTTTCTACTGTTTTACGTCCTTTGC
**PEX13 Gsc PEX5 W4**	chimera	TAACAAAGCCCGAAAGGAAGCTGAGTTG	ACCGCGACCGATCGCTGGTATGATGAATATCATCCGGAAGAAGATTAACAAAGCCCGAAAGGAAGCTGAGTTG	TGAGCTTTCTACTGTTTTACGTCCTTTGC	ATCTTCTTCCGGATGATATTCATCATACCAGCGATCGGTCGCGGTGCTGCCTCCACCGCCTGAGC
**PEX14 NTD**	1-104	ATGGCTAGCAGCGAACAGGCC	GAACAGATTGGTGGCAATGGCTAGCAGCGAACAGGCC	ACTACCCGCCGGAGAATACGG	TTTCGGGCTTTGTTAACTACCCGCCGGAGAATACGG
**PEX14 NTD long**	1-113	ATGGCTAGCAGCGAACAGGCC	GAACAGATTGGTGGCAATGGCTAGCAGCGAACAGGCC	CGCCAGTGCGCCATAATCG	TTTCGGGCTTTGTTACGCCAGTGCGCCATAATCG
**PEX5 W0**	1-76	ATGGCAATGCGGGAGCTG	GAACAGATTGGTGGCATGGCAATGCGGGAGCTG	GGACACAAGGGGTGCATTCTG	TTTCGGGCTTTGTTAGGACACAAGGGGTGCATTCTG

Supplementary Table 6

Primer list for cloning PEX13 C-term variants into pETM13S vector.

Construct	Boundaries	Forward short	Forward tail	Reverse short	Reverse tail
**PEX13 C-term**	YGG 351-403	TACGGAGGACAACAGCAGTCATTCACCAATCC	GAACAGATTGGTGGCTACGGAGGACAACAGCAGTCATTCACCAATCC	TTTGGTACCTCACAAATCCTGTTTTTCACCG	TTTCGGGCTTTGTTATTTGGTACCTCACAAATCCTGTTTTTCACCG
**PEX13 C-term FxxxF to AAAAA**	YGG 351-403	GTTGAGACTAATAAAGTGCCGGTGG	GCGGCGGCGGCGGCGGTTGAGACTAATAAAGTGCCGGTGGC	CGCGGCTTCTTGCTCGTC	CGCCGCCGCCGCCGCGGCGGCTTCTTGCTCGTC
**PEX13 C-term FxxxF to PEX5 W4**	YGG 351-403	GTTGAGACTAATAAAGTGCCGGTGG	TGGTACGACGAATACGTTGAGACTAATAAAGTGCCGGTGGC	CGCGGCTTCTTGCTCGTC	GTATTCGTCGTACCACGCGGCTTCTTGCTCGTC
**PEX13 SH3 V275Y_V276E**	261-346	GCACGTGCGGAATATGATTTCGC	GATGATCACTATGAAGCACGTGCGGAATATGATTTCGC	TTCTCCTGAGGCCCAGTTGATG	TTCATAGTGATCATCTTCTCCTGAGGCCC
**PEX13 SH3 K304G**	261-346	CCAAAAGTGCGCGGCTG	TTAGGCGAGCAGCAGCCAAAAGTGC	TGCCAAGTTCAGCATGTCCC	CTGCTGCTCGCCTAATGCCAAGTTCAGCATGTCCC
**PEX13 CTD I333C**	261-403	CTGGGTAAACGCAAAGGACG	AATTACGTCAAATGCCTGGGTAAACGCAAAGGACG	CGCTGGAATCAGGCCTGTAG	GCATTTGACGTAATTCGCTGGAATCAGG
**PEX13 CTD A376C**	261-403	TTCGTTGAGACTAATAAAGTGCCGG	TGCTTCGAATCCGTCTTCGTTGAGAC	GGCTTCTTGCTCGTCCAAAG	GACGGATTCGAAGCAGGCTTCTTGCTCGTCCAAAG

Supplementary Table 7

Primer list for cloning into bi-cistronic vector pIRES2 - GFP.

Time	Boundaries	Forward short	Forward tail	Reverse short	Reverse tail
**pEB100 pIres 2**	backbone	GCGGGCCCGGGATCC	GCAGTCGACGGTACCGCGGGCCCGGGATCC	CTGAGTCCGGTAGCGCTAGC	GCTTGAGCTCGAGATCTGAGTCCGGTAGCGCTAGC
**PEX13 FL**	1-403	ATGGCGTCCCAGCCG	ATCTCGAGCTCAAGCATGGCGTCCCAGCCG	TTAAAGATCTTGCTTTTCTCCATCTTTCCC	GGTACCGTCGACTGCTTAAAGATCTTGCTTTTCTCCATCTTTCCC
**PEX13 ΔCTR**	1-264	GCAGTCGACGGTACCGC	GAAGTAACAGACAGCTAAGCAGTCGACGGTACCGCGG	ATCACTGTGAGTAGACAATAGTTTCC	TTAGCTGTCTGTTACTTCATCACTGTGAG
**PEX13 ΔC-term**	1-346	GCAGTCGACGGTACCGC	GAAGTAACAGACAGCTAAGCAGTCGACGGTACCGCGG	ACTTGATTCCACCGTTTTCCTAC	GGTACCGTCGACTGCACTTGATTCCACCGTTTTCCTAC
**PEX13 ΔSH3**	1-261, 346-403	ACGGTGGAATCAAGTAAAGTTTCCAAGC	GGCGGTGGAGGCTCTACGGTGGAATCAAGTAAAGTTTCCAAGC	GCTGTCTGTTACTTCATCACTGTGAG	AGAGCCTCCACCGCCGCTGTCTGTTACTTCATCACTGTGAG
**PEX3 FxxxF / 5A**	1-403 +mutation	GTTGAAACTAATAAGGTTCCAGTTGCAC	GCAGCCGCTGCAGCTGTTGAAACTAATAAGGTTCCAGTTGCAC	GGCAGCTTCCTGTTCATCCAAAG	AGCTGCAGCGGCTGCGGCAGCTTCCTGTTCATCCAAAG
**PEX3 FxxxF / W4**	1-403 +mutation	GTTGAAACTAATAAGGTTCCAGTTGCAC	TGGTACGACGAATACGTTGAAACTAATAAGGTTCCAGTTGCAC	GGCAGCTTCCTGTTCATCCAAAG	GTATTCGTCGTACCAGGCAGCTTCCTGTTCATCCAAAG
**PEX13 V275Y/ V276E**	1-403	GCCAGAGCAGAATATGATTTTGCTGC	GATGACCATTACGAAGCCAGAGCAGAATATGATTTTGCTGC	CTCACCACTTGCCCAGTTGATG	TTCGTAATGGTCATCCTCACCACTTGCC
**PEX13 K304G**	1-403	CCCAAAGTGCGTGGTTGGC	CTCGGAGAACAACAACCCAAAGTGCGTGG	AGCTAAGTTCAGCATATCACCAGC	TTGTTGTTCTCCGAGAGCTAAGTTCAGCATATCACCAGC
**PEX13 I333C**	1-403	CTTGGCAAAAGAAAAGGTAGGAAAACG	AATTATGTCAAATGCCTTGGCAAAAGAAAAGGTAGGAAAACG	CGCAGGTATAAGTCCTGTTGTTTGG	GCATTTGACATAATTCGCAGGTATAAGTCCTGTTG
**PEX13 A376C**	1-403	TTTGTTGAAACTAATAAGGTTCCAGTTGCAC	TGCTTTGAATCTGTTTTTGTTGAAACTAATAAGGTTCCAGTTGCAC	AGCTTCCTGTTCATCCAAAGAATCAGC	AACAGATTCAAAGCAAGCTTCCTGTTCATCCAAAGAATCAGC

Supplementary Table 8

Primer list for cloning into bi-cistronic vector pIRES2 - GFP.

Time	Bound-aries	Forward short	Forward tail	Reverse short	Reverse tail
**pETM24 His-Gb1-GFP**	backbone	ATGGTGAGCAAGGGCGAG	AGCGGAGGATCGGGTGGAATGGTGAGCAAGGGCGAG	CTCAGAACCACTGCCAGATCC	GGCGCCCTGAAAATAAAGATTCTCAG
**pETM24 His-Gb1-mCherry**	backbone	ATGTCAAAAGGTGAAGAACTGTTCACTG	AGCGGAGGATCGGGTGGAATGTCAAAAGGTGAAGAACTGTTCACTG	CTCAGTAGTGGGATGTCGTAATC	GGCGCCCTGAAAATAAAGATTCTCAG
**PEX5 mCherry**	1-330	ATGGCAATGCGGGAGCTG	AATCTTTATTTTCAGGGCGCCATGGCAATGCGGGAGCTG	GTTCTCCTCCTCAAACTGGTACCC	TCCACCCGATCCTCCGCTGTTCTCCTCCTCAAACTGGTACCC
**PEX14 GFP**	1-104	ATGGCTAGCAGCGAACAGGC	AATCTTTATTTTCAGGGCGCCATGGCTAGCAGCGAACAGGC	ACTACCCGCCGGAGAATACG	TCCACCCGATCCTCCGCTACTACCCGCCGGAGAATACG

Supplementary Table 9

Sequences of human SH3 domains for MSA.

Accession Code (UniProtKB)	Protein name	Aligned sequence
Q92968	PEX13_HUMAN Peroxisomal membrane protein PEX1	SINWASGEDDHVVARAEYDFAAVSEEEISF RAGDMLNLALKEQQPKVRGWLLASLDGQ TTGLIPANYVKILGKRKGRKTVESSK
P80667	PEX13_YEAST Peroxisomal membrane protein PAS20	ASQGNGSEPIDPSKLEFARALYDFVPENPE MEVALKKGDLMAILSKKDPLGRDSDWWK VRTKNGNIGYIPYNYIEIIKRRKKIEHVD
P12931	SRC_HUMAN Proto-oncogene tyrosine-protein kinase Src	QRAGPLAGGVTTFVALYDYESRTETDLSF KKGERLQIVNNTEGDWWLAHSLSTGQTG YIPSNYVAPSDSIQAEEWYF
P46108	CRK_HUMAN Adapter molecule crk OS=Homo sapiens	SGVILRQEEAEYVRALFDFNGNDEEDLPFK KGDILRIRDKPEEQWWNAEDSEGKRGMIP VPYVEKYRPASASVSAL
Q8N157	AHI1_HUMAN Jouberin OS=Homo sapiens	HQVDTAPTVVALYDYTANRSDELTIHRGDI IRVFFKDNEDWWYGSIGKGQEGYFPANHV ASETLYQELPPEIKERSPP
O43639	NCK2_HUMAN Cytoplasmic protein NCK2	MTEEVIVIAKWDYTAQQDQELDIKKNERL WLLDDSKTWWRVRNAANRTGYVPSNYVE RKNSLKKGSLV
Q8TE68	ES8L1_HUMAN Epidermal growth factor receptor kinase substrate 8-like protein 1	PQLESETAGKWVLCNYDFQARNSSELSVK QRDVLEVLDDSRKWWKVRDPAGQEGYVP YNILTPYPGPRLHHSQ
O75791	GRAP2_HUMAN GRB2-related adapter protein 2	PVQLQAAGRVRWARALYDFEALEDDELG FHSGEVVEVLDSSNPSWWTGRLHNKLGLF PANYVAPMT
P27986	P85A_HUMAN Phosphatidylinositol 3-kinase regulatory subunit alpha	MSAEGYQYRALYDYKKEREEDIDLHLGDI LTVNKGSLVALGFSDGQEARPEEIGWLNG YNETTGERGDFPGTYVEYIGRKKISPPTPKP
Q5TCZ1	SPD2A_HUMAN SH3 and PX domain-containing protein	GADATAEPMILEQYVVVSNYKKQENSELS LQAGEVVDVIEKNESGWWFVSTSEEQGW VPATYLEAQNGTRDDSDINT

References

1. Ashkenazy H*, et al.* ConSurf 2016: an improved methodology to estimate and visualize evolutionary conservation in macromolecules. *Nucleic Acids Research* **44**, W344-W350 (2016).
2. Celniker G*, et al.* ConSurf: Using Evolutionary Data to Raise Testable Hypotheses about Protein Function. *Israel Journal of Chemistry* **53**, 199-206 (2013).
3. Ashkenazy H, Erez E, Martz E, Pupko T, Ben-Tal N. ConSurf 2010: calculating evolutionary conservation in sequence and structure of proteins and nucleic acids. *Nucleic Acids Research* **38**, W529-W533 (2010).
4. Shi X*, et al.* Structural recognition mechanisms between human Src homology domain 3 (SH3) and ALG-2-interacting protein X (Alix). *FEBS letters* **586**, 1759-1764 (2012).
