# Supplementary material for: Modulation of peroxisomal import by the PEX13 SH3 domain and a proximal FxxxF binding motif
Source: Nat Commun. 2024 Apr 18;15:3317. doi: 10.1038/s41467-024-47605-w (PMC11024197; doi:10.1038/s41467-024-47605-w)
Supplement: Supplementary file 2 — Reporting Summary [file 41467_2024_47605_MOESM2_ESM.pdf]

nature portfolio

Corresponding author(s): Michael Sattler, Ralf Erdmann

Last updated by author(s): Mar 20, 2024

Reporting Summary

Nature Portfolio wishes to improve the reproducibility of the work that we publish. This form provides structure for consistency and transparency in reporting. For further information on Nature Portfolio policies, see our Editorial Policies and the Editorial Policy Checklist.

Statistics

For all statistical analyses, confirm that the following items are present in the figure legend, table legend, main text, or Methods section.

n/a	Confirmed	
☐	☒	The exact sample size (*n*) for each experimental group/condition, given as a discrete number and unit of measurement
☐	☒	A statement on whether measurements were taken from distinct samples or whether the same sample was measured repeatedly
☐	☒	The statistical test(s) used AND whether they are one- or two-sided<br>*Only common tests should be described solely by name; describe more complex techniques in the Methods section.*
☒	☐	A description of all covariates tested
☐	☒	A description of any assumptions or corrections, such as tests of normality and adjustment for multiple comparisons
☐	☒	A full description of the statistical parameters including central tendency (e.g. means) or other basic estimates (e.g. regression coefficient) AND variation (e.g. standard deviation) or associated estimates of uncertainty (e.g. confidence intervals)
☐	☒	For null hypothesis testing, the test statistic (e.g. *F*, *t*, *r*) with confidence intervals, effect sizes, degrees of freedom and *P* value noted<br>*Give P values as exact values whenever suitable.*
☒	☐	For Bayesian analysis, information on the choice of priors and Markov chain Monte Carlo settings
☒	☐	For hierarchical and complex designs, identification of the appropriate level for tests and full reporting of outcomes
☒	☐	Estimates of effect sizes (e.g. Cohen's *d*, Pearson's *r*), indicating how they were calculated

Our web collection on statistics for biologists contains articles on many of the points above.

Software and code

Policy information about availability of computer code

Data collection	NMR: Topspin 3.7; X-ray: SLS or EMBL in-house data collection pipeline, ITC: MicroCal 1.21 (Malvern Analytics); SEC-SLS: Omnisec 11.01 (Malvern Analytics);Modelling: Maestro 12.8 (Schrodinger suite); Microscopy: Zen 3.6 blue edition (Zeiss); Immunoblot: LI-COR Acquisition Software 1.2 (LI-COR Biosciences), Image Studio 5.2 (LI-COR Biosciences)
Data analysis	NMR: NMRPipe, CcpNMR Analysis 2.4.2; X-ray: XDS built 20220110, ccp4i2 software package v.1.1.12; ITC: MicroCal 1.21 (Malvern Analytics); SEC-SLS: Omnisec 11.01 (Malvern Analytics); Modelling: Maestro 12.8 (Schrodinger suite); Microscopy: ZEN 2.3 blue edition (Zeiss), Prism 10.0.0 (GraphPad), ImageJ 1.54d; Immunoblot: ImageJ 1.54f, Empiria Studio 2.3 (LI-COR Biosciences), Prism 10.0.0 (GraphPad),

For manuscripts utilizing custom algorithms or software that are central to the research but not yet described in published literature, software must be made available to editors and reviewers. We strongly encourage code deposition in a community repository (e.g. GitHub). See the Nature Portfolio guidelines for submitting code & software for further information.

Data

Policy information about availability of data

All manuscripts must include a data availability statement. This statement should provide the following information, where applicable:

- Accession codes, unique identifiers, or web links for publicly available datasets
- A description of any restrictions on data availability
- For clinical datasets or third party data, please ensure that the statement adheres to our policy

UniProtKB : PEX13 = Q92968, PEX14 = O75381, PEX5 = P50542
NMR chemical shift assignment data: PEX13 SH3-CTR (BMRB 51336)
Crystallographic data (PDB): PEX13 SH3: PDB (7Z0I); PEX13 SH3-FxxxF complex: PDB (7Z0J); PEX13 SH3-PEX5-W4 complex: PDB (7Z0K)

Human research participants

Policy information about studies involving human research participants and Sex and Gender in Research.

Reporting on sex and gender	N/A
Population characteristics	N/A
Recruitment	N/A
Ethics oversight	N/A

Note that full information on the approval of the study protocol must also be provided in the manuscript.

Field-specific reporting

Please select the one below that is the best fit for your research. If you are not sure, read the appropriate sections before making your selection.

☒ Life sciences ☐ Behavioural & social sciences ☐ Ecological, evolutionary & environmental sciences

For a reference copy of the document with all sections, see nature.com/documents/nr-reporting-summary-flat.pdf

Life sciences study design

All studies must disclose on these points even when the disclosure is negative.

Sample size	For microscopy images, the sample size for all cell lines was based on at least 15 images, usually showing more than 100 cells in total. Only for slower growing cells (dSH3-CTR, dCTR, dSH3, IA/CC), the sample size was smaller but comprised at least 45 cells.
Data exclusions	No data are excluded from this manuscript
Replication	All experiments were performed with three replicates, all attempts were successful.
Randomization	For immunoblot analysis of the digitonin fractionation experiments, several SDS gels had to be used due to the number of samples to be analyzed. For randomization, the different cell lines were loaded onto the gels randomly and in different combinations for each biological replicate.
Blinding	N/A. For analysis of microscopy images, the risk of bias was minimized by automated determination of the Pearson Coefficient.

Reporting for specific materials, systems and methods

We require information from authors about some types of materials, experimental systems and methods used in many studies. Here, indicate whether each material, system or method listed is relevant to your study. If you are not sure if a list item applies to your research, read the appropriate section before selecting a response.

Materials & experimental systems

n/a	Involved in the study
☐	☒ Antibodies
☐	☒ Eukaryotic cell lines
☒	☐ Palaeontology and archaeology
☒	☐ Animals and other organisms
☒	☐ Clinical data
☒	☐ Dual use research of concern

Methods

n/a	Involved in the study
☒	☐ ChIP-seq
☒	☐ Flow cytometry
☒	☐ MRI-based neuroimaging

Antibodies

Antibodies used

PMP70 (Invitrogen PA1-650), Thiolase (Sigma Aldrich HPA007244), GAPDH (Proteintech 60004-1-Ig), GFP (Santa Cruz sc-9996), PEX13 (Proteintech 26649-1-AP) Rabbit PEX5 (Proteintech 12545-1-AP), Chicken PEX14 (doctoral thesis Lena Brühl, Ruhr-University Bochum, Germany), Mouse PEX5 (Ruhr-University Bochum)

Validation

Mouse PEX5 is described in reference 65.
Chicken antiserum raised against the N-terminal domain of purified human His6-PEX14(aa1-80) was validated by applying lysates of HEK 293 cells, T-REx 293 cells and corresponding PEX14KO cell lines to immunoblot analysis. A single band at the known molecular weight for the target (57 kDa) was observed in wildtype but not in KO cell lines.
Other antibodies are commercially available and were validated by the manufacturer.

Eukaryotic cell lines

Policy information about cell lines and Sex and Gender in Research

Cell line source(s)

T-REx(TM)293 (Invitrogen, USA) cells were modified user CRISPR/Cas9 as described in reference 35 to generate a T-REx 293 PEX13KO cell line

Authentication

Described in reference 35.

Mycoplasma contamination

Cell lines were not recently tested for Mycoplasma contamination

Commonly misidentified lines
(See ICLAC register)

N/A
